# The use of artificial intelligence in assessing fever in the returning traveller: A scoping review

**DOI:** 10.1371/journal.pdig.0001742

**Published:** 2026-10-01

**Authors:** Bhavya Gandhi, Leo Morjaria, Imeth Illamperuma, Meha Bhatt, Andrew Kapoor

**Affiliations:** 1 Michael G. DeGroote School of Medicine, McMaster University, Hamilton, Ontario, Canada; 2 Division of General Internal Medicine, Michael G. DeGroote School of Medicine, McMaster University Medical Center, Hamilton, Ontario, Canada; 3 Division of Infectious Diseases, Michael G. DeGroote School of Medicine, McMaster University Medical Center, Hamilton, Ontario, Canada; University of Nottingham, UNITED KINGDOM OF GREAT BRITAIN AND NORTHERN IRELAND

## Abstract

Fever in the returning traveller is a challenging presentation that requires a wide array of clinical expertise and draws on complex medical decision-making. Recent advancements in artificial intelligence have shown it can be a helpful clinical support tool in this context. This scoping review, conducted in accordance with the Preferred Reporting Items for Systematic Reviews and Meta-Analyses extension for Scoping Reviews guidelines and registered on the Open Science Framework (https://doi.org/10.17605/OSF.IO/BSPKX), aimed to identify the current state of artificial intelligence use in assessing fever in the returning traveller. Here we describe the three eligible studies evaluating three distinct AI approaches identified in the literature. These heterogenous studies reported promising performance on study-specific endpoints in controlled settings. Given the lack of external validation, narrow populations studied, and absence of real-world implementation or user acceptance testing, further research is needed to explore this technology in a real-world clinical setting. The limited available evidence suggests that AI may have future potential to support assessment of fever in the returning traveller; however, clinical utility, safety, and real-world effectiveness remain unestablished. Large language models represent an emerging area of interest but were not evaluated in the studies included in this review.

## Author summary

We wanted to understand how artificial intelligence has been used to help clinicians assess fever in people who become ill after international travel. This clinical problem is difficult because the list of possible infections is broad and depends on travel destination, exposure history, symptoms, and test results. We reviewed the published literature and found three eligible studies. These studies described different approaches: a rule-based expert system to generate a ranked list of possible diagnoses, a machine-learning model to predict malaria risk, and a model using immune markers to help distinguish between infections. All three studies suggested that artificial intelligence could support clinical assessment in controlled settings. We also found that all studies were conducted in hospital-based populations. None of the included studies reported real-world clinical implementation or user acceptability testing, and external validation was limited. We believe this review shows that artificial intelligence is a promising but early area in travel-related fever assessment. More research, including external validation and real-world implementation studies, is needed before these tools can be confidently adopted in routine clinical care.

## Introduction

Fever in the returning traveller (FRT) is a common but often challenging presentation given its broad differential diagnosis [1]. It is estimated that 25% of Canadians returning from travel may experience a travel-related illness [2,3]. Prompt and accurate assessment of these presentations is essential as they can be associated with diseases such as malaria, dengue, typhoid, and TB [4]. Globalization, increased rates of post pandemic travel, as well as recent emerging diseases such as the COVID-19, Zika, and Chikungunya have highlighted the growing complexity of diagnosing infectious diseases in returning travelers [4,5].

Currently, febrile illness in the returning traveller is assessed through a detailed history, physical examination, and pathogen-specific investigations [2]. This approach relies on front-line clinicians having a high level of clinical expertise in travel and tropical medicine, emerging diseases, and general infectious diseases.

Artificial intelligence (AI), though in its infancy as a clinical tool, is already integrated into electronic medical record platforms to assist with documentation, summarizing complex conversations and developing clinical plans [6]. It has demonstrated potential in clinical diagnostics and risk prediction, with effective emergent tools spanning from breast cancer diagnosis through mammograms analysis algorithms to detection of pneumonia on chest radiography [7]. AI models are uniquely positioned to synthesize large amounts of data for clinicians including data such as regional outbreak data, pathogen specific geography, or incubation periods of pathogens [8]. This information is essential for accurate clinical diagnosis but can be difficult and time-consuming to access during brief routine patient care. Such technology could be leveraged to guide clinical decision-making by generating a comprehensive differential diagnosis, for example, allowing clinicians to use their clinical judgment to determine the final diagnosis.

Developing AI tools to assess FRT can support clinical decision-making to overcome the need to integrate vast amounts of clinical and non-clinical information into assessments. Ultimately this may provide the avenue to develop new tools to aid in the timely diagnosis and management of FRT. Despite its potential, there are currently few studied clinical applications of AI within infectious diseases, and the extent of its use specifically in FRT remains unclear.

This scoping review aims to examine the current use of AI in assessing FRT. By synthesizing the available evidence, this review offers important insights into the current uses of AI in this context, its potential benefits, limitations, and key considerations.

## Methods

### Ethics Statement

This scoping review synthesized data from previously published studies. Research ethics board review and informed consent were therefore not required.

This scoping review was structured in adherence to the PRISMA-ScR (Preferred Reporting Items for Systematic Reviews and Meta-Analyses extension for Scoping Reviews) guidelines (See S1 Table). This study was registered within the Open Science Framework (OSF) database (https://doi.org/10.17605/OSF.IO/BSPKX). A scoping review methodology was chosen to map the available evidence in an emerging and heterogenous field, given initial literature searches demonstrated a lack of recent reviews addressing our research question [9].

### Literature search

An initial search of MEDLINE (Ovid) was undertaken to identify articles. This was used to identify keywords and terms to develop the full search strategy. The search strategy was peer-reviewed by a university research librarian. Our search strategy encompassed four concepts: artificial intelligence, infectious diseases, fever, and returning travellers. The search design involved identification of synonyms for these concepts as well as Boolean operators. No search filters were used to limit the search results. The search strategy used is available through OSF as part of the published study protocol.

The databases searched include MEDLINE (Ovid), EMBASE (Ovid), Cochrane Central Register of Controlled Trials (CENTRAL), and Web of Science databases. Sources of unpublished studies and gray literature were not included. Searches included records published in the databases from their inception up the time of data collection which was February 07, 2025.

### Eligibility criteria

This scoping review considered both experimental and quasi-experimental study designs including randomized controlled trials and non-randomized controlled trials. Observational studies including prospective and retrospective cohort studies, case-control studies and cross-sectional studies were included.

Inclusion criteria: Studies including adult patients who are returning travellers from any length of foreign travel from their home country with an infectious disease(s) as a cause of fever. Studies must use of a form of AI explicitly in the context of infectious disease assessment, including history, physical, diagnosis, clinical decision support, risk stratification, and/or management to be included. For this scoping review, AI was defined broadly to include both rule-based expert systems and machine learning approaches, which were grouped for mapping purposes but interpreted as distinct methods. Studies must have outcomes related to diagnostic accuracy, predictive value, efficiency, clinical utility, or impact of AI in clinical practice. These outcomes did not have to pertain directly to the use of AI in clinical assessment. Studies must have occurred in healthcare, research, or academic settings to be included.

Exclusion criteria: Studies focusing on non-travel-related fever or non-infectious causes of fever and studies with populations not defined as returning travellers were excluded. Studies including only paediatric patients were also excluded. Studies that did not specifically apply AI to the assessment of FRT or were situated in non-healthcare-related settings (ex. Training programs) were excluded.

### Study selection

Covidence was used to manage the data outputted from the structured search strategy. Duplicates were removed and titles and abstracts were screened by two independent reviewers based on the inclusion/exclusion criteria. A full text review was conducted next with two independent reviewers. Reasons for exclusion were recorded. Disagreements between reviewers were resolved through discussion and a third reviewer where agreement could not be reached. The methodology is outlined in Fig 1 as a PRISMA diagram.

**Fig 1:**
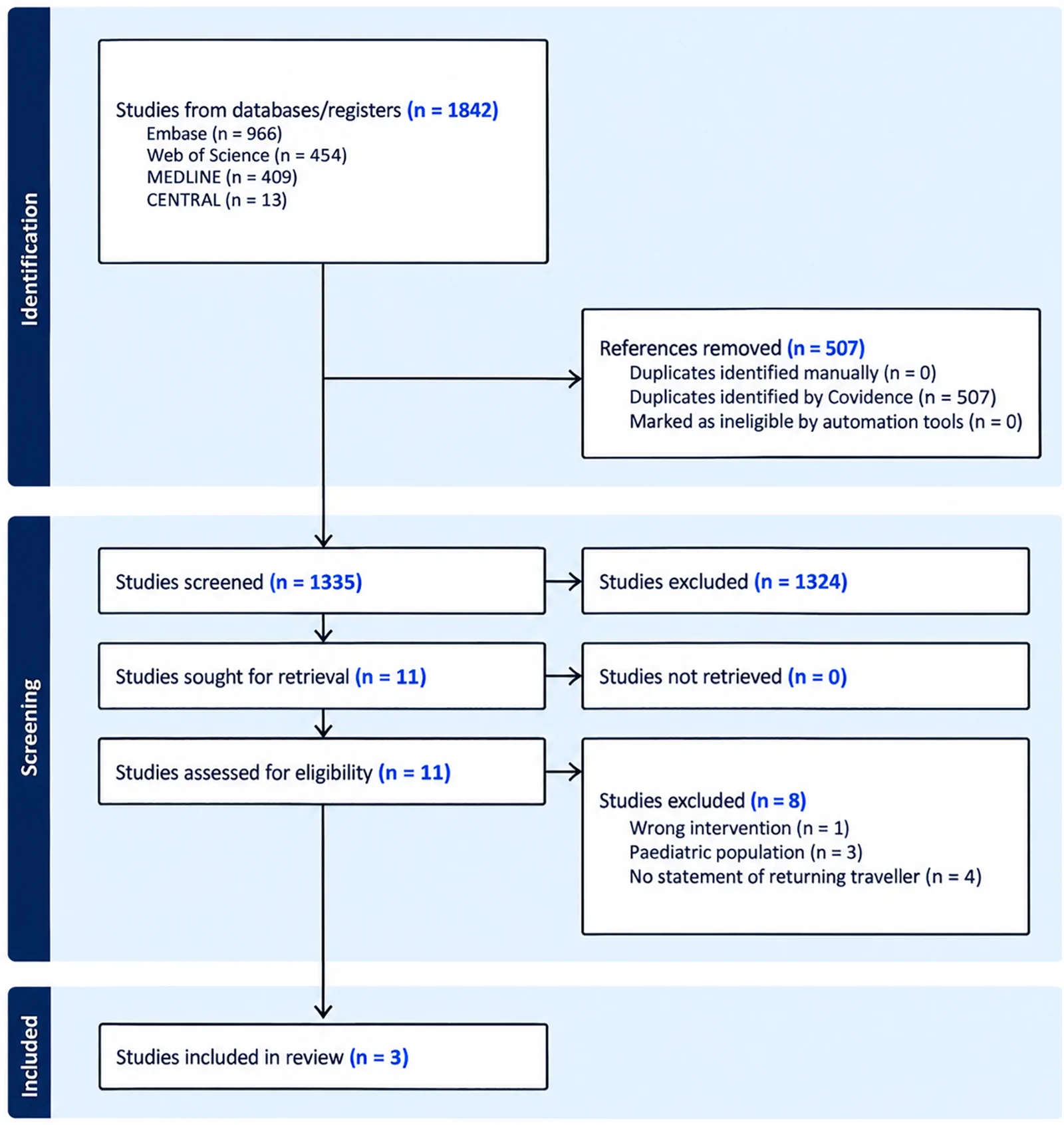
Preferred Reporting Items for Systematic Reviews and Meta-Analyses extension for Scoping Reviews (PRISMA-ScR) flow chart.

### Data extraction

Data was extracted from the selected citations by independent reviewers using the Covidence platform. The data extracted included: authors; year of publication, article title, country in which the study conducted, study methods, study objective, study design, start and/or end date, if provided, study funding sources, possible conflicts of interest for study authors, population description, clinical setting, inclusion criteria, exclusion criteria, total number of participants, AI method(s), method architecture (if any), function of model (intended use), input data; output data (how does the output data support assessment of FRT), user acceptance; qualitative information on user acceptance of deployed models where reported, key qualitative and quantitative results reported, key contributions, author-reported limitation, and author-reported future directions. The full extracted data is provided in the supplementary appendix (S1 Table). Extracted data was synthesized using a narrative approach. A quality or risk of bias assessment was not conducted, given the scoping review was conducted using PRISMA-ScR methodology.

## Results

1335 unique articles were identified from the literature search. Following title and abstract screening, 1324 articles were excluded, and 11 articles underwent full-text review by two reviewers independently. 8 articles were excluded. 1 included the wrong intervention, 3 included primarily pediatric populations, and 4 had no specific consideration of returning travellers. Three articles met inclusion criteria. Two were prospective cohort studies and one was an exploratory observational study. The studies were based in Belgium (n = 1), Sweden (n = 1), and one was an international multi-centre study including Spain, Belgium, Switzerland (n = 1).

The mean number of study participants was 354 (standard deviation [SD]: 355). Studies selected for patients over the age of 18 (n = 2) and unspecified (n = 1). All three studies selected for patients with febrile illness described as fever (≥37.5°C) or symptoms of fever (n = 1), fever >38°C (measured or self-reported) (n = 1), and unspecified (n = 1). Similarly, all three articles included patients that recently returned from international travel within the past year (n = 1), the past two months (n = 1) and unspecified (n = 1). No explicit exclusion criteria were listed for any of the studies. All studies were conducted in hospital settings (n = 3) and all were multi-year studies including multiple seasons (n = 3).

### Model characteristics

Three unique architectures were used: GIDEON rule-based system (n = 1), boosting algorithms (i.e. XGBoost) (n = 1), a Boruta feature selection model, and random forest model with 5-fold and 10-fold cross-validation. (n = 1). The rule-based system provided an output of a ranked list of differential diagnoses, each with an associated probability [10]. The boosting algorithm, XGBoost (reduced to a six-feature model), outputted a binary classification of suspected malaria listed as ‘yes’ or ‘no’ [11]. The random forest model generated disease classification per patient [12].

The GIDEON rule-based expert system intended to provide a list of differential diagnoses to support the management of febrile illness in patients returning from recent travel. While area under the curve (AUC) was not listed for the GIDEON rule-based expert system, a diagnostic accuracy (correct diagnosis in top 5 outputs) of 64% in febrile cases with confirmed or probable infectious etiology was achieved. This was higher in cases of malaria, rickettsial, and dengue, reaching over 80%. Accuracy declines were noted with cases of amebic liver abscess and *Plasmodium falciparum malaria*, and in cases of greater than three clinical finding inputs [10].

The MALrisk algorithm (XGBoost reduced to a six-feature model) aimed to identify febrile patients at high risk of malaria in non-endemic settings. The MALrisk model achieved an AUC of 0.95 with a sensitivity of 100% (95% CI: 96–100) and specificity of 72% (95% CI: 68–75) in the test set and an AUC of 0.98 (F1 score of 0.78) in the training set [11].

The random forest model intended to differentiate specific infections in febrile returning travellers using immune signatures. This model achieved an AUC of 0.97 with some variation across models targeted towards specific pathologies such as bacterial gastroenteritis. AUC was seen to improve with addition of lab markers to the model. Generally, sensitivities and specificities were greater than 0.87 and 0.72, respectively [12].

None reported on user acceptance including data examining end-user engagement or real-world pilot testing. Data was not provided on clinician satisfaction, usability, or real-world complications/applicability.

### Model variables

The specific data inputted in each model is listed in Supplementary S2 Table. Common variables inputted include epidemiological findings including location of travel and demographic data, specific symptoms, and laboratory values like platelet count and bilirubin. Other variables included were antimalarial chemoprophylaxis intake and plasma cytokine/chemokine levels, generally used due to the use-case of the associated model.

## Discussion

AI methods may facilitate the simultaneous analysis of multiple clinical and epidemiological variables and therefore have potential utility in diagnostic decision support. This review identified three current published applications of AI applied to the assessment of FRT. Given the complexity of this presentation, and the many associated variables, AI may have a future role in supporting assessment; however, the current evidence remains preliminary. This systematic literature search identified three heterogenous applications of AI currently studied for use in the assessment of FRT. The included studies employed a rule-based model, a gradient boosting algorithm, and random forest model. Varying endpoints led models to produce unique outputs such as a list of differential diagnosis, identifying risk for suspected malaria, or differentiation of specific infections, respectively. These models reported promising performance in achieving their study-specific endpoints. The included studies demonstrate that distinct AI approaches can generate clinically interpretable outputs from structured inputs; however, reproducibility across populations, institutions, and clinical environments has not been established [10]. All three models highlight the potential utility and viability of AI support in clinical evaluation of patients returning from travel with consistency given standard inputs, but do not establish real-world effectiveness, safety, or scalability.

### Model architecture

Each model utilized core principles of AI in clinical decision support, generally centered around decision trees, to produce their respective outputs. However, these architectures are not methodologically equivalent, particularly because the rule-based expert system relies on predefined logic while the machine learning models learn statistical patterns from data. The rule-based model employs a decision tree-like approach by following a set of defined rules to produce an outcome. For example, an input provided will traverse through a decision tree similar to decision trees used by clinicians. The gradient boosting algorithm is fundamentally a compilation of many decisions-trees to identify errors in previous predictions and produce the best possible output. These are developed through sequential training to identify such errors as opposed to defining rules. Finally, a random forest model uses multiple decision trees to produce a large group of outputs that are then compiled by the model to produce a final output.

The intricacies of the input data used as a basis for the models varied significantly. For example, the random forest model designed to differentiate between specific infections used plasma cytokine/chemokine levels as one of its variables, a value not often used in standard clinical practice [12]. Due to the limited literature on such levels and the lack of existing models leveraging these values, this model generated novel interpretations for applications in a clinical setting. Indirectly, this model provided insight into the correlation between specific pathologies and proteomics - information that may become more available and useful in clinical practice within the context of such models. For example, an elevated IL10, sCD25, and sTNFRI, and reduced sCD40L were found to be associated with malaria [12]. While not part of routine clinical testing for malaria, these markers have been well studied in the basic science realm, and within the context of a comprehensive clinical model may lead to more accurate prediction scores than currently available.

None of the models used greater than six categories of variables as input data. This may be a reflection of the context limitations of existing AI architectures that begin to see a plateau or even decline in performance when given too many parameters to correlate [11]. The MALrisk model identifies reduction to six variables from an initial boosting algorithm as potentially supportive of future implementation by reducing the number of required inputs. Fewer variables may reduce data entry and computational requirements; however, the included study did not evaluate whether this translated into greater clinician uptake, scalability, or implementation success [11]. As the capabilities advance, it is possible that more variables will be included. These advancements may enable newer algorithms to improve the accuracy of their outputs, though this requires empirical validation in FRT.

Similar to input data, the nature of output data varied between models. The GIDEON rule-based system, developed a list of differential diagnoses and included ‘why-not’ and ‘compare’ functions that provided further explanations for the outputs produced [10]. Alternatively, the MALrisk model produced a binary output (yes/no) to identify a suspected malaria case [11]. From the perspective of model functions, inputs and outputs both require interpretation, analysis and synthesis of the data provided, however, they do hold differing practical implications. A detailed list of differential diagnoses with included explanations can provide higher-level clinical decision-making support for healthcare providers aiming to expand their diagnoses and management plans. Alternatively, a binary output can support short-term actions such as empiric treatments, reporting, testing, and transfer to appropriate facilities. Both output models may have applicability for ruling in/out high-risk conditions (e.g., malaria) and capturing broader differential diagnoses to not miss less common presentations or pathogens. Both applications highlight the importance of catering model outputs tailored to a clinically useful endpoint.

### Model limitations

Despite the results of the models, there remain limitations of their applicability. Firstly, models were not used in clinical pilot studies and there was no end-user engagement resulting in the lack of data surrounding clinical applicability, clinician satisfaction, and real-world errors. This limits the ability to identify the feasibility of model integration in frontline healthcare settings that may experience unique constraints like time, delayed charting, and limited history from patients. The mean number of study participants was 354 (standard deviation [SD]: 355) with most articles identifying a lack of diversity within the study population, limiting the generalizability of the application of these models. External validation was also limited, further restricting conclusions about performance outside the original study settings. As with many AI models in healthcare, the opportunity to use them in pilot studies remains limited [6]. This stems from multiple systems-based limitations like the lack of healthcare policies on AI use as well as little infrastructure to support its integration (ex. technical support team with training in AI clinical decision support systems). Clinicians continue to have concerns regarding the implications of AI on clinical judgement and workflow, often concerned about errors that may be produced [13]. For example, MALrisk had a misclassification rate of 28% for non-malarial febrile patients [11]. The GIDEON rule-based system missed 31 total diagnoses, 11 of which were severe or life-threatening such as *Plasmodium falciparum malaria* [10]. Beyond this, limitations exist intrinsically within the architecture of these models. The variables chosen and the specific data inputted can be biased based on the data collector, inputter, or designer of these models. For example, the specificity of the travel region inputted can provide different levels of context to an AI model. Stating ‘sub-Saharan Africa’ may develop different correlations compared to stating a specific country. Study design in AI model development also comes with risk of bias. For example, while the selection of a boosting algorithm to develop MALrisk is largely appropriate, similar results may be achieved with other forms of AI, especially considering recent evolution.

### Advancements in artificial intelligence

Importantly, none of the three studies included in this review evaluated large language models (LLMs); therefore, the following discussion reflects emerging opportunities for future research rather than evidence identified through this review. With the recent advent of highly capable, publicly accessible large language models (LLMs), the production of models to support clinical decision-making has become increasingly simple. Recent literature has shown that LLMs can perform at the same level or better than physicians when providing written responses to healthcare questions spanning multiple domains [14]. There exists significant potential for clinical applications of models to support clinical decision-making in the context of FRT by leveraging this technology, but this remains speculative in FRT. To address concerns around patient safety and medical errors, the use of virtual patients produced using LLMs provides a controlled, reproducible, high-fidelity environment for pilot testing [15]. Beyond this, true pilot testing in real-world scenarios is essential to the further development of this technology.

### Clinical applications

Development of AI models to assess FRT may offer opportunities to support clinicians who must integrate substantial clinical and epidemiological information. Clinicians face significant levels of burnout driven by increasing levels of charting, electronic data review, and administrative work [16]. AI models that support clinical synthesis for decision-making are a well-positioned tool clinicians may be able to leverage in making real-time decisions in the future if validated and safely integrated into clinical workflows. These studies suggest that relatively simple models can generate potentially useful clinical outputs in controlled settings; however, none of the included studies established improvements in patient outcomes or clinical decision-making. Such models could potentially be adapted for use across different healthcare settings, but cost-effectiveness, scalability, and implementation were not evaluated in the included studies [14,15]. In a primary care setting, such models may be able to aid clinicians in identifying the most likely cause of FRT and guide potential next steps. These models may help inform when referral is warranted as well as information to provide [17,18]. Overall, this represents a potential future application, but effects on clinician workload, treatment timeliness, resource utilization, and patient outcomes require prospective evaluation.

### Future directions

Given the application of AI clinical support models remain in their early stages, further research is needed to explore their applicability and assess their limitations in the context of assessing FRT. Despite our broad literature search, only three applications were identified in this setting. This may be partly due to the relative infancy of AI in medicine as well as hesitancy for adoption, including healthcare policy limitations, lack of existing infrastructure and clinician concerns surrounding reliability [13]. Studies using a wider variety of AI methods, such as LLMs, applied in controlled simulation environments followed by externally validated prospective studies could help establish whether these approaches have clinical value in FRT. Further advances in LLM capabilities may warrant future investigation for clinical decision support, although their role in FRT remains untested [14]. For example, LLMs with these capabilities may be able to produce a list of differential diagnoses and key next steps for a management plan based on a history, physical examination and brief investigations inputted by a clinician.

### Limitations

Several limitations exist in this scoping review, primarily, the lack of literature in this context limits the ability to draw broader conclusions on the use of AI to assess FRT. Additionally, as a scoping review, this study was designed to map available evidence rather than formally compare effectiveness. Secondly, due to the heterogeneity of the studies included, such as differing models, statistical measures, and variable endpoints, a true comparison between performance could not be accurately assessed. The rapid evolution of AI technologies may also affect the relevance of individual models over time and complicate comparisons across studies conducted in different technological eras. This scoping review simply maps the current landscape and synthesizes the early developments in this field. While limited by the available evidence, this review establishes a foundation for future research on the role of AI in assessing FRT, rather than evidence of clinical readiness.

## Conclusion

FRT remains a diagnostically challenging presentation demanding focused clinical expertise and skill for accurate diagnosis and efficient management. AI-based decision-support approaches may eventually assist clinicians managing this complex presentation, but their effects on diagnostic accuracy, clinician workload, and patient outcomes have not yet been established in real-world FRT care. This scoping review identified three studies evaluating distinct AI approaches for the assessment of FRT, each reporting promising performance on study-specific outcomes in controlled settings. These findings should be considered preliminary and demonstrate proof-of-concept rather than clinical readiness, effectiveness, or scalability. Further research incorporating, external validation, prospective clinical evaluation, user acceptance testing, implementation assessment, and safety evaluation, will be essential to understand whether and how these tools can be safely integrated into clinical care.

### Key points

Artificial intelligence has been used for assessment of fever in the returning traveller with models reporting promising performance won study-specific outcomes in controlled settings, though evidence remains limited and heterogenous.

## Supporting information

S1 Table Preferred Reporting Items for Systematic reviews and Meta-Analyses extension for Scoping Reviews (PRISMA-ScR) Checklist Completed PRISMA-ScR checklist with corresponding manuscript page numbers. (PDF)

S2 Table Characteristics of included studies Characteristics of included studies, including study design, population, artificial intelligence methods, key findings, limitations, and future directions. (XLSX)
