# Supplementary material for: The use of artificial intelligence in assessing fever in the returning traveller: A scoping review
Source: PLOS Digit Health. 2026 Oct 1;5(10):e0001742. doi: 10.1371/journal.pdig.0001742 (PMC13630189; doi:10.1371/journal.pdig.0001742)
Supplement: Supplementary file 2 [file pdig.0001742.s004.docx]

**RESPONSE TO EDITOR AND REVIEWERS**

**PLOS Digital Health**

**Manuscript ID:** PDIG-D-26-00556

**Title:** The use of artificial intelligence in assessing fever in the returning traveller: a scoping review

**Corresponding author:** Dr. Andrew Kapoor

Dear Dr. Turner, Dr. Lu, Dr. Celi, and Reviewers,

Thank you for the thoughtful and constructive evaluation of our manuscript. We are grateful for the opportunity to revise the work. We have carefully considered each comment and revised the manuscript to improve the alignment of our conclusions with the limited and heterogeneous evidence base, clarify the conceptual distinction between rule-based expert systems and machine-learning approaches, strengthen the discussion of limitations, and more clearly separate the findings of the review from future-oriented discussion. A marked-up manuscript showing all changes and a clean revised manuscript are included with the resubmission.

Our point-by-point responses are provided below. Reviewer comments are reproduced in italics, followed by our responses and a summary of the corresponding revisions.

# Reviewer #1

“My main concern is that the discussion and conclusion sometimes go beyond what the evidence can support. The review includes only three heterogeneous studies, all in hospital-based settings, with no real-world implementation studies, no user-acceptance data, and limited external validation. Because of this, some claims about AI being an effective, scalable, or broadly applicable clinical support tool in this setting feel too strong and should be stated more cautiously.”

**Response:** We agree. The manuscript has been revised throughout to use more cautious language and to distinguish promising model performance in controlled study settings from demonstrated clinical effectiveness. We now emphasize that the three included studies do not establish real-world effectiveness, safety, scalability, cost-effectiveness, or readiness for routine implementation.

**Changes made:** The Abstract, Discussion, Clinical Applications, Limitations, and Conclusion were revised to qualify claims and foreground the narrow populations, hospital-based settings, limited external validation, absence of implementation studies, and lack of user-acceptance data. In particular, the Discussion now explicitly states that the included models “do not establish real-world effectiveness, safety, or scalability,” and the concluding sections frame the evidence as an early foundation for future study rather than evidence of clinical readiness.

“A second concern is conceptual clarity. One included tool is a rule-based expert system, while the others are machine-learning approaches, and these are not equivalent methods. The paper would be stronger if it explained more clearly why these systems were grouped together under artificial intelligence and what the implications of that choice are.”

**Response:** We agree and have clarified this distinction. The review used a broad mapping definition of artificial intelligence in order to capture the full range of computational decision-support approaches applied to fever in returning travellers. However, the revised manuscript now explicitly states that rule-based expert systems and machine-learning models were grouped for mapping purposes while being interpreted as methodologically distinct approaches.

**Changes made:** The Eligibility Criteria section now defines AI broadly while noting that rule-based and machine-learning methods were treated as distinct. The Model Characteristics and Model Architecture sections were expanded to explain that the rule-based system applies predefined logic, whereas the machine-learning models learn statistical patterns from data. The implications of this methodological heterogeneity are also acknowledged in the Discussion and Limitations.

“The manuscript should also better separate what was actually found in the review from future-oriented discussion about large language models and other emerging tools, since those points are more speculative than evidence-based in the current review.”

**Response:** We agree. We have more clearly separated the evidence identified by the scoping review from discussion of emerging technologies. Large language models are now presented as a future research direction rather than as an intervention evaluated by the included studies.

**Changes made:** The Advancements in Artificial Intelligence and Future Directions sections now explicitly state that applications of large language models in this specific clinical context remain speculative and untested. Statements describing the results of the review are limited to the three included approaches: a rule-based expert system, a gradient-boosting model, and a random-forest model.

“Overall, this is a useful scoping review on a relevant topic, but it needs revision to bring the conclusions into closer alignment with the limited and heterogeneous evidence base… and strengthening the discussion of limitations.”

**Response:** Thank you. We have strengthened the limitations throughout the revised manuscript and aligned the conclusions more closely with the available evidence.

**Changes made:** The revised manuscript now gives greater prominence to the small number of eligible studies, heterogeneity in model architecture and endpoints, hospital-based populations, limited population diversity, lack of direct implementation testing, absent user-acceptance data, and limited external validation. The Limitations section also clarifies that the scoping-review design maps the available evidence rather than providing a comparative estimate of effectiveness.

# Reviewer #2

“The abstract structure did not follow the formal structure of highlighting background, aim, methods, results and conclusion.”

**Response:** Thank you. We revised the Abstract so that the background, objective and methods, results and interpretation, and conclusion are clearly presented in sequence. The revised Abstract also more directly reports the small number of included studies and the principal evidence limitations.

**Changes made:** The Abstract was reorganized into clear conceptual components and revised to include the review registration, the three major applications identified, the limited external validation and narrow populations studied, and the need for real-world evaluation.

“Finishing the introduction, no one citation was given for the background research or any literature review on the subject of the manuscript.”

**Response:** Thank you for identifying this area for clarification. We revised the Introduction to better connect the broader literature on AI-supported clinical decision-making with the specific evidence gap in fever among returning travellers. We also clarified at the end of the Introduction that the extent of AI use in this narrowly defined setting remains uncertain and therefore warrants evidence mapping.

**Changes made:** The background discussion and final rationale in the Introduction were refined, and the purpose of the review is now stated more directly. The Methods also explains that preliminary searches identified no recent review addressing the specific research question.

“The recurring trend of using scoping review is a limitation of this study methodology when compared to systematic review. It is easier to perform and more flexible in dealing with the available research materials.”

**Response:** We agree that the methodological choice should be justified and its limitations acknowledged. A scoping-review approach was selected because the field is emerging, the evidence is sparse, and the included studies use heterogeneous architectures, inputs, outputs, and endpoints. The purpose was therefore to map the range and characteristics of available evidence rather than to estimate a pooled effect or directly compare effectiveness.

**Changes made:** The Methods now provides an explicit rationale for selecting a scoping-review design. The Limitations section further states that this methodology was intended to map available evidence rather than formally compare effectiveness, and that study heterogeneity prevented a meaningful direct performance comparison.

“The number of cited references is one of the smallest lists that would be encountered in reviewing.”

**Response:** We appreciate this observation. The narrow reference base reflects the highly specific research question and the identification of only three eligible primary studies. We reviewed the reference list for completeness and retained literature directly relevant to returning-traveller fever, the included computational approaches, implementation considerations, and emerging AI methods, while avoiding citations that did not materially support the manuscript.

**Changes made:** The contextual discussion was reviewed to ensure that key clinical, methodological, implementation, and future-oriented statements were supported by relevant references. The manuscript also now more explicitly explains that the small evidence base is itself a central finding of the review.

“Figure 1. One of the most elegant, to-the-point and easy to follow figures ever reviewed. It delivered the message and no single item or step is vague or worth skipping.”

**Response:** Thank you for this positive assessment. We retained the PRISMA-ScR flow diagram and its concise presentation.

“Considering the new advances in AI and the advent of LLMs, this is an important section to add, and it is a future projection that is worth considering.”

**Response:** Thank you. We retained this future-oriented discussion while revising it to make clear that large language models were not evaluated in the included studies and that their application to fever in returning travellers remains speculative and requires empirical validation.

**Changes made:** The Advancements in Artificial Intelligence section now explicitly distinguishes future possibilities from the review findings and emphasizes the need for controlled evaluation, external validation, safety assessment, and real-world pilot testing.

“Again, in the ‘Future Directions’ is a leading predictive opinion that despite the infancy of AI in medicine and the small number of applications studied, it would be expected that these applications will be considered pioneering experiments.”

**Response:** Thank you. We retained the Future Directions section because it helps define a research agenda for this emerging field, while moderating the wording so that future applications are framed as possibilities that require validation rather than expected clinical developments.

“In the limitation sections, ‘a true comparison between performance could not be accurately assessed.’ This is the most important quote. The idea of comparing performance of different applications and models… would be the basis for future evaluation of performance of AI in medicine.”

**Response:** We agree. We retained and strengthened this point. Because the included studies differed in architecture, intended use, input variables, output definitions, and reported performance measures, direct comparison would be inappropriate. The revised manuscript identifies standardized evaluation and comparative validation as priorities for future research.

**Changes made:** The Limitations section now more clearly states that heterogeneity prevented formal comparison, and the Discussion emphasizes the need for external validation and clinically meaningful, standardized evaluation in future studies.

“I would like to congratulate the authors on this work… My recommendation for this manuscript is to be published.”

**Response:** We sincerely thank the reviewer for the encouraging assessment and constructive comments.

# Editorial and Journal Requirements

Although the decision letter indicated that technical items do not need to be addressed point-by-point in this response, we confirm the following for completeness:

- **Ethics Statement:** A complete Ethics Statement now appears at the beginning of the Methods section under the required subheading.
- **Supporting Information:** The supplementary tables have been removed from the main manuscript file and will be uploaded separately as Supporting Information. Legends are listed after the references in the revised manuscript.
- **Data Availability Statement:** The submission form will include the statement: “All data are in the manuscript and/or supporting information files.”
- **Revised files:** A marked-up manuscript with tracked changes and a clean unmarked manuscript are included with the resubmission.

**Closing statement**

We thank the editors and reviewers again for their careful evaluation. We believe the revisions have improved the conceptual clarity, methodological transparency, and balance of the manuscript, and have brought the conclusions into closer alignment with the available evidence. We hope that the revised manuscript is now suitable for publication in PLOS Digital Health.

Sincerely,

Andrew Kapoor, MD, MPH, MSc, FRCPC
On behalf of all authors
