# Supplementary material for: The use of artificial intelligence in assessing fever in the returning traveller: A scoping review
Source: PLOS Digit Health. 2026 Oct 1;5(10):e0001742. doi: 10.1371/journal.pdig.0001742 (PMC13630189; doi:10.1371/journal.pdig.0001742)
Supplement: Supplementary file 3 [file pdig.0001742.s005.docx]

**RESPONSE TO EDITOR AND REVIEWERS**

**PLOS Digital Health**

**Manuscript ID:** PDIG-D-26-00556R1

**Title:** The use of artificial intelligence in assessing fever in the returning traveller: a scoping review

**Corresponding author:** Dr. Andrew Kapoor

Dear Dr. Turner, Dr. Lu, Dr. Celi, and Reviewer,

Thank you for the thoughtful and constructive evaluation of our revised manuscript. We are grateful for the opportunity to make these additional revisions. We have carefully considered the reviewer’s comments and revised the manuscript to bring the interpretation and conclusions into closer alignment with the small and heterogeneous evidence base, clearly distinguish the review findings from discussion of emerging large language models, and strengthen the reporting of data availability and references. A marked-up manuscript showing all changes and a clean revised manuscript will accompany the resubmission.

Our point-by-point responses are provided below. Reviewer comments are reproduced in italics, followed by our responses and a summary of the corresponding revisions.

# Reviewer #1

“This is a useful scoping review on an important and emerging topic. The review is well organized and clearly shows that only a small number of studies have evaluated AI for fever in returning travellers. I also found the comparison of the different AI approaches helpful.”

**Response:** We sincerely thank the reviewer for this encouraging assessment and for recognizing the value of mapping the limited evidence in this emerging area.

“My main suggestion is to make some parts of the discussion and conclusion more cautious. Since only three studies were included, with limited external validation and no real-world implementation or user-acceptance testing, claims about scalability, clinical impact, or improved patient outcomes should be presented as future possibilities rather than established findings.”

**Response:** We agree. The manuscript has been revised throughout to distinguish promising performance on study-specific outcomes in controlled settings from demonstrated clinical effectiveness. We now emphasize that the three included studies do not establish real-world clinical utility, safety, scalability, cost-effectiveness, improved patient outcomes, or readiness for implementation.

**Changes made:** The Key Points and Abstract were revised to describe three eligible studies evaluating three distinct AI approaches, remove claims of established effectiveness or reproducibility, and explicitly identify limited external validation, narrow study populations, and the absence of implementation and user-acceptance testing. The Abstract conclusion now states that clinical utility, safety, and real-world effectiveness remain unestablished. The Discussion now characterizes the evidence as preliminary and states that reproducibility across populations, institutions, and clinical environments has not been established. Claims about the reduced-input MALrisk model, scalability, clinician uptake, and implementation success were qualified. The Clinical Applications section now treats effects on clinician workload, treatment timeliness, resource use, and patient outcomes as questions for prospective evaluation rather than demonstrated benefits. The Future Directions, Limitations, and Conclusion sections were also revised. The conclusion now presents the studies as preliminary proof-of-concept and identifies external validation, prospective clinical evaluation, user-acceptance testing, implementation assessment, and safety evaluation as requirements before clinical integration.

“I also suggest clearly separating the discussion of newer tools such as large language models from the evidence actually included in the review.”

**Response:** We agree and have more clearly separated the evidence identified by the scoping review from future-oriented discussion of emerging technologies. Large language models are now presented only as a potential direction for future research, not as an approach evaluated by any included study.

**Changes made:** The Advancements in Artificial Intelligence section now opens with an explicit statement that none of the three included studies evaluated large language models. The section and the Future Directions discussion now frame LLM applications in fever among returning travellers as speculative and untested, and propose controlled simulation followed by externally validated prospective studies before any assessment of clinical value.

“A few small reporting issues, including the brief Data Availability Statement and duplicated reference, should also be corrected.”

**Response:** We agree and have corrected both reporting issues.

**Changes made:** The Data Availability Statement was expanded to confirm that all review data are available within the manuscript and Supporting Information, identify Supplementary Table 2 as containing the complete consensus extraction data, and provide the DOI for the registered protocol and search strategy on the Open Science Framework. The reference list was reviewed and the duplicated reference was removed.

“Overall, I recommend minor revision.”

**Response:** We thank the reviewer for the recommendation and believe the revisions have strengthened the accuracy, balance, and transparency of the manuscript.

We thank the editors and reviewer again for their careful evaluation. We believe the revisions more accurately reflect the preliminary nature of the evidence, clearly distinguish the included studies from future research opportunities, and improve the manuscript’s reporting. We hope that the revised manuscript is now suitable for publication in PLOS Digital Health.

Sincerely,

Andrew Kapoor, MD, MPH, MSc, FRCPC
On behalf of all authors
