# Supplementary material for: The use of artificial intelligence in assessing fever in the returning traveller: A scoping review
Source: PLOS Digit Health. 2026 Oct 1;5(10):e0001742. doi: 10.1371/journal.pdig.0001742 (PMC13630189; doi:10.1371/journal.pdig.0001742)
Supplement: Peer review history

# Peer review: Decision Letter 0

**DOI:** 10.1371/journal.pdig.0001742.r001

**Authors:** Henry Horng-Shing Lu, Alexander Turner

22 May 2026

Response to Reviewers Revised Manuscript with Track Changes Manuscript **Journal Requirements:**

1. Please ensure that your Ethics Statement is available in its entirety at the beginning of your Methods section, under a subheading 'Ethics Statement'.

2. We notice that your supplementary table are included in the manuscript file. Please remove them and upload them with the file type 'Supporting Information'. Please ensure that each Supporting Information file has a legend listed in the manuscript after the references list.

3. Please provide a complete Data Availability Statement in the submission form, ensuring you include all necessary access information or a reason for why you are unable to make your data freely accessible. If your research concerns only data provided within your submission, please write "All data are in the manuscript and/or supporting information files" as your Data Availability Statement.

**Additional Editor Comments (if provided):** **Reviewers' Comments:**

**Comments to the Author**

1. Does this manuscript meet PLOS Digital Health’s publication criteria?

Reviewer #1: Yes

Reviewer #2: Yes

**********

2. Has the statistical analysis been performed appropriately and rigorously?

Reviewer #1: N/A

Reviewer #2: Yes

**********

3. Have the authors made all data underlying the findings in their manuscript fully available (please refer to the Data Availability Statement at the start of the manuscript PDF file)?

The PLOS Data policy

Reviewer #1: Yes

Reviewer #2: Yes

**********

4. Is the manuscript presented in an intelligible fashion and written in standard English?

Reviewer #1: Yes

Reviewer #2: Yes

**********

Reviewer #1: This manuscript addresses a timely and relevant topic. Fever in the returning traveller is a difficult clinical problem, and reviewing how artificial intelligence has been used in this setting is worthwhile. I also appreciate that the review follows a scoping review framework, is registered on OSF, and clearly shows that only a small number of studies currently exist in this area.

The main strength of the paper is that it identifies a genuine gap in the literature. The review found only three eligible studies, and these studies represent different approaches, including a rule-based expert system, a malaria prediction model, and an immune-marker-based classification model. This is useful descriptive work and provides a starting point for future research.

My main concern is that the discussion and conclusion sometimes go beyond what the evidence can support. The review includes only three heterogeneous studies, all in hospital-based settings, with no real-world implementation studies, no user-acceptance data, and limited external validation. Because of this, some claims about AI being an effective, scalable, or broadly applicable clinical support tool in this setting feel too strong and should be stated more cautiously.

A second concern is conceptual clarity. One included tool is a rule-based expert system, while the others are machine-learning approaches, and these are not equivalent methods. The paper would be stronger if it explained more clearly why these systems were grouped together under artificial intelligence and what the implications of that choice are. The manuscript should also better separate what was actually found in the review from future-oriented discussion about large language models and other emerging tools, since those points are more speculative than evidence-based in the current review.

Overall, this is a useful scoping review on a relevant topic, but it needs revision to bring the conclusions into closer alignment with the limited and heterogeneous evidence base. I would recommend major revision, with particular attention to toning down overextended claims, clarifying the conceptual framing of the included AI methods, and strengthening the discussion of limitations.

Reviewer #2: Thanks for the opportunity to review this manuscript. It is pioneering in its idea. The work is disadvantaged with the limited scope of the medical field that it is examining. However, it is a courageous choice by the authors to explore this field with regard to AI implementation. One would imagine that it is a limited field and the efforts exerted in reviewing hundreds or thousands of literature documents to end up with three studies to put in the published review. It is an inspiring piece of work. I have bulleted down few comments because I did not find much to add or to comment about.

• The abstract structure did not follow the formal structure of highlighting background, aim, methods, results and conclusion.

• Finishing the introduction, no one citation was given for the background research or any literature review on the subject of the manuscript.

• The recurring trend of using scoping review is a limitation of this study methodology when compared to systematic review. It is easier to perform and more flexible in dealing with the available research materials.

• The number of cited references is one of the smallest lists that would be encountered in reviewing.

• “Figure 1.” One of the most elegant, to-the-point and easy to follow figures ever reviewed. It delivered the message and no single item or step is vague or worth skipping.

• Considering the new advances in AI and the advent of LLMs, this is an important section to add, and it is a future projection that is worth considering. It would soon be possible that these applications would just be taken for granted and this section could be cited as a future prediction.

• Again, in the “Future Directions” is a leading predictive opinion that despite the infancy of AI in medicine and the small number of applications studied, it would be expected that these applications will be considered pioneering experiments.

• In the limitation sections, “a true comparison between performance could not be accurately assessed.” This is the most important quote. The idea of comparing performance of different applications and models and it would be the bases for future evaluation of performance of AI in medicine.

• I would like to congratulate the authors on this work. I don’t have much to add to the limitations that were discussed transparently by the authors.

• My recommendation for this manuscript is to be published.

Best wishes,

Yasser

**********

what does this mean?). If published, this will include your full peer review and any attached files.

**Do you want your identity to be public for this peer review?**  If you choose “no”, your identity will remain anonymous but your review may still be made public.

For information about this choice, including consent withdrawal, please see our Privacy Policy

Reviewer #1: **Yes:**  Sudhakavya Bodapati Venkata

Reviewer #2: **Yes:**  Yasser Abdullah

**********

**Figure resubmission:**

**Reproducibility:**

To enhance the reproducibility of your results, we recommend that authors of applicable studies deposit laboratory protocols in protocols.io, where a protocol can be assigned its own identifier (DOI) such that it can be cited independently in the future. Additionally, PLOS ONE offers an option to publish peer-reviewed clinical study protocols. Read more information on sharing protocols at https://plos.org/protocols?utm_medium=editorial-email&utm_source=authorletters&utm_campaign=protocols

# Author comment: Author response to Decision Letter 1

**DOI:** 10.1371/journal.pdig.0001742.r002

24 Jul 2026

Attachment Submitted filename: Response_to_Reviewers_PLOS_Digital_Health.docx

# Peer review: Decision Letter 1

**DOI:** 10.1371/journal.pdig.0001742.r003

**Authors:** Henry Horng-Shing Lu, Alexander Turner

13 Aug 2026

Response to Reviewers Revised Manuscript with Track Changes Manuscript **Journal Requirements:**

1. We note that you have included your Figures within the body of your manuscript. Please remove the Figures from the body of your manuscript and upload them as separate Figure files in .tif or .eps format.

For more information about figure files please see our guidelines:  https://journals.plos.org/digitalhealth/s/figures

https://journals.plos.org/digitalhealth/s/figures#loc-file-requirements

**Additional Editor Comments (if provided):**

"This is a useful scoping review on an important and emerging topic. The review is well organized and clearly shows that only a small number of studies have evaluated AI for fever in returning travellers. I also found the comparison of the different AI approaches helpful.

My main suggestion is to make some parts of the discussion and conclusion more cautious. Since only three studies were included, with limited external validation and no real-world implementation or user-acceptance testing, claims about scalability, clinical impact, or improved patient outcomes should be presented as future possibilities rather than established findings.

**Reviewers' Comments:**

**Comments to the Author**

Reviewer #1: (No Response)

**********

publication criteria?

Reviewer #1: Yes

**********

3. Has the statistical analysis been performed appropriately and rigorously?

Reviewer #1: N/A

**********

4. Have the authors made all data underlying the findings in their manuscript fully available (please refer to the Data Availability Statement at the start of the manuscript PDF file)?

The PLOS Data policy

Reviewer #1: Yes

**********

5. Is the manuscript presented in an intelligible fashion and written in standard English?

Reviewer #1: Yes

**********

**Reviewer #1:**  This is a useful scoping review on an important and emerging topic. The review is well organized and clearly shows that only a small number of studies have evaluated AI for fever in returning travellers. I also found the comparison of the different AI approaches helpful.

My main suggestion is to make some parts of the discussion and conclusion more cautious. Since only three studies were included, with limited external validation and no real-world implementation or user-acceptance testing, claims about scalability, clinical impact, or improved patient outcomes should be presented as future possibilities rather than established findings.

I also suggest clearly separating the discussion of newer tools such as large language models from the evidence actually included in the review. A few small reporting issues, including the brief Data Availability Statement and duplicated reference, should also be corrected. Overall, I recommend minor revision.

**********

what does this mean?). If published, this will include your full peer review and any attached files.

**Do you want your identity to be public for this peer review?**  If you choose “no”, your identity will remain anonymous but your review may still be made public.

For information about this choice, including consent withdrawal, please see our Privacy Policy

Reviewer #1: **Yes:**  Sudhakavya Bodapati Venkata

**********

**Figure resubmission:**

**Reproducibility:**

To enhance the reproducibility of your results, we recommend that authors of applicable studies deposit laboratory protocols in protocols.io, where a protocol can be assigned its own identifier (DOI) such that it can be cited independently in the future. Additionally, PLOS ONE offers an option to publish peer-reviewed clinical study protocols. Read more information on sharing protocols at https://plos.org/protocols?utm_medium=editorial-email&utm_source=authorletters&utm_campaign=protocols

# Author comment: Author response to Decision Letter 2

**DOI:** 10.1371/journal.pdig.0001742.r004

6 Sep 2026

Attachment Submitted filename: Response to Reviewers - 2.docx

# Editor report: Decision Letter 2

**DOI:** 10.1371/journal.pdig.0001742.r005

**Authors:** Henry Horng-Shing Lu, Alexander Turner

7 Sep 2026

The use of artificial intelligence in assessing fever in the returning traveller: a scoping review

PDIG-D-26-00556R2

Dear Dr. Kapoor,

We are pleased to inform you that your manuscript 'The use of artificial intelligence in assessing fever in the returning traveller: a scoping review' has been provisionally accepted for publication in PLOS Digital Health.

Before your manuscript can be formally accepted you will need to complete some formatting changes, which you will receive in a follow-up email from a member of our team.

Please note that your manuscript will not be scheduled for publication until you have made the required changes, so a swift response is appreciated.

IMPORTANT: The editorial review process is now complete. PLOS will only permit corrections to spelling, formatting or significant scientific errors from this point onwards. Requests for major changes, or any which affect the scientific understanding of your work, will cause delays to the publication date of your manuscript.

If your institution or institutions have a press office, please notify them about your upcoming paper to help maximize its impact. If they'll be preparing press materials, please inform our press team as soon as possible -- no later than 48 hours after receiving the formal acceptance. Your manuscript will remain under strict press embargo until 2 pm Eastern Time on the date of publication. For more information, please contact digitalhealth@plos.org.

Thank you again for supporting Open Access publishing; we are looking forward to publishing your work in PLOS Digital Health.

Best regards,

Alexander Turner

Academic Editor

PLOS Digital Health

***********************************************************

**Additional Editor Comments (if provided):**

**Reviewer Comments (if any, and for reference):**
